# Exercise may delay cognitive decline in Chinese older adults: a causal inference for ordered multi-categorical exposures with a Mendelian randomization approach

**DOI:** 10.1038/s41598-024-59326-7

**Published:** 2024-06-06

**Authors:** Lizhen Han, Yi Zeng, Tao Huang, Jinzhu Jia

**Affiliations:** 1 Center for Clinical and Epidemiologic Research, Beijing An Zhen Hospital, Capital Medical University, Beijing Institute of Heart, Lung and Blood Vessel Diseases, Beijing, 100029 China; 2 Center for Healthy Aging and Development Studies, National School of Development, Peking University, Beijing, 100191 China; 3 Center for Study of Aging and Human Development and Geriatrics Division, School of Medicine, Duke University, Durham, NC USA; 4 Department of Epidemiology & Biostatistics, School of Public Health, Peking University, No. 38, Xueyuan Road, Haidian District, Beijing, 100191 China; 5 Center for Intelligent Public Health, Academy for Artificial Intelligence, Peking University, Beijing, 100871 China; 6 Department of Biostatistics, School of Public Health, Peking University, No. 38, Xueyuan Road, Haidian District, Beijing, 100191 China; 7 Center for Statistical Science, Peking University, Beijing, 100871 China

**Keywords:** Casual inference, Ordered multi-categorical exposures, Cognitive impairment, Mendelian randomization, Exercise, Cognitive ageing, Genetic association study, Risk factors, Statistics, Epidemiology, Genetics research

## Abstract

The cognitive problems are prominent in the context of global aging, and the traditional Mendelian randomization method is not applicable to ordered multi-categorical exposures. Therefore, we aimed to address this issue through the development of a method and to investigate the causal inference of cognitive-related lifestyle factors. The study sample was derived from the Chinese Longitudinal Healthy Longevity Survey, which included 897 older adults aged 65 + . This study used genome-wide association analysis to screen genetic loci as instrumental variables and innovatively combined maximum likelihood estimation to infer causal associations between ordered multi-categorical exposures (diet, exercise, etc.) and continuous outcomes (cognitive level). The causal inference method for ordered multi-categorical exposures developed in this study was simple, easy to implement, and able to effectively and reliably discover the potential causal associations between variables. Through this method, we found a potential positive causal association between exercise status and cognitive level in Chinese older adults ($\widehat{\beta }$ = 1.883, 95%*CI* 0.182–3.512), in which there was no horizontal pleiotropy (*p* = 0.370). The study provided a causal inference method applicable to ordered multi-categorical exposures, that addressed the limitations of the traditional Mendelian randomization method.

## Introduction

Cognitive-related health issues are increasingly prominent against the backdrop of global aging. As of 2019, there were already more than 50 million people living with dementia worldwide, with an average of one new case of dementia every three seconds. More than 75 million people will suffer from dementia by 2030^1,2^. China is a country with a huge aging population. Currently, the number of older adults aged 60 years and above in China has reached 264.02 million, accounting for 18.70% of the population^3^.

We have explored some factors (e.g., diet, activity status, etc.) associated with cognitive impairment in the Chinese older adults through our previous studies^4,5^. In order to better clarify whether there is some causal association, we therefore investigated the issue with the help of Mendelian randomization (MR). It is a method that uses genetic variation as the instrumental variable (IV) to make causal inferences about the effect of exposure on outcome^6–8^. However, this method has limitations when the exposure is ordered multi-categorical variables as such. Briefly, if an observed multi-level categorical exposure (i.e., intensities of exercise) is the manifestation of an underlying continuous exposure (i.e., motivation to exercise) passing certain cut-off points, the observed level of category may be stable even if the latent exposure has changed, because the latter haven’t crossed one of the cut-offs. This may violate the assumption of exclusivity of the instrumental variable^9–11^. In this scenario, disregarding the relationship between a latent, invisible continuous exposure and its external, visible categorical manifestation will bias the MR/IV estimation of effect size (if the latter is a strong stepwise mediator), or even confound the claim of casual association (if the latter is mostly a surrogate)^12^.

In view of the above issues, we aimed to develop and test an analytical pipeline of Mendelian randomization, tailed to ordered multi-categorical exposures and to further explore potential causal associations between cognitive levels and their influencing factors in the Chinese older adults.

## Materials and methods

### Data sources

Data for this study were obtained from the Chinese Longitudinal Healthy Longevity Survey (CLHLS) database (1998–2018)^13,14^. This was the largest and most extensive cohort of older adults in China, encompassing more than 40,000 older adults in total. The project mainly collected information on the socio-demographic characteristics, lifestyles, and health status of participants through a questionnaire (self-report). A total of 908 surviving older adults aged 65 years or older (based on the age of the participants when they first participated in the survey) were selected from individuals who participated in both the questionnaire and whole genome sequencing (WGS). Meanwhile, we collated their baseline information. After quality control (QC)^15^ of the genetic data, a total of 897 older adults aged 65–110 years were included (no missing data), with a male to female ratio of approximately 1:2.74.

### Cognition

Individual cognitive data were collected and calculated by the Mini-mental State Examination (MMSE). This scale provides a comprehensive assessment of the individual's general ability, reactivity, attention and calculation, recall, language, comprehension and self-coordination. The total score is 30, with higher scores indicating a better cognitive status^16^.

### Living habits

Based on the results of our previous studies conducted on CLHLS^4,5^, we selected those factors that had some association with individual cognitive level for inclusion in this study. The variables in this section included individual drinking status, exercise status, dietary habits, and activity participation at their baseline. The drinking status were categorized by drinking (including abstainers) and never drinking. Exercise status was similarly divided into two categories according to exercise and never exercise. Diet and activity participation were recorded in terms of frequency, in descending order of "frequently", "occasionally" and "rarely/never", with higher values representing lower frequency. Other variables and detailed descriptions were given in Additional file 1.

### Gene

Genetic data were obtained from the Longevity Study (CLHLS) conducted by Prof. Yi Zeng.^17^ The data were generated from whole genome sequencing and genotyped by Illumina humanomnizhhua-8 BeadChips. The chip was created by selecting optimally tagged single nucleotide polymorphisms (SNPs) from all three phases of the the International HapMap Project as well as from the Thousand Genomes Project. It covered 900 015 SNPs, including 600 000 common variants (Minor allele frequency, MAF ≥ 5%), 290 000 rare variants (MAF < 5%), and 10 000 SNPs present only in Chinese and other Asian populations. In addition to directly sequenced SNPs, the project also used IMPUTE software (version 2) to infer genotypes for SNPs with MAF ≥ 0.01 (The 1000 Genomes Project as a reference), representing approximately 85.38%^17^.

We performed quality control on the raw genetic data and examined the population stratification (multidimensional scaling, MDS)^15^. A total of 3 240 266 SNPs were included in this study with genotype data covering autosomes 1–22. The genotype of each SNP was recorded in the form of an additive model (i.e., 0, 1, 2). Chromosome location information was obtained from hg19.

### Genome-wide association study

Genome-wide association study (GWAS)^15^ was conducted by plink 1.9 for individual cognitive level, living habits and potential confounders (e.g., educational status and stroke/CVD^5^). Among them, continuous variables were analyzed using linear regression, while categorical variables were performed through logistic regression. The covariates included were age, sex, ethnicity, and 10 dimensions representing stratified characteristics of the population obtained by multidimensional scaling. Subsequently, the Quantile–Quantile plot (Q-Q plot) and Manhattan plot were plotted based on the results obtained from GWAS.

### Instrumental variables

Instrumental variables (IVs) were selected from independent loci that were significant in GWAS. In order to better validate the generalizability and stability of our method, we relaxed the selection criterion of p-value to "close to significance" when selecting instrumental variables. Based on the results of GWAS, independent and significant SNPs (*p* < 1 × 10^–5^) satisfying the three assumptions of instrumental variables^8^ were selected as IVs for each exposure after screening to exclude those loci that were associated with known confounders.

Specifically, the sub-routine groups significant SNPs into genome segments, that is, clumps, that are independently and significantly associated with the outcome, each indexed by one of the highly significant SNPs within. Each new clump is iteratively isolated by (1) greedily search the next most significant SNP with p-value no larger than 1e^-5^ (via –clump-p1 0.00001) but not yet enclosed in any existing clumps, that is, the so-called index variant of the soon to be new clump, then (2) adding variants within close proximity to the index, both in terms of physical units (+ /− 500 KB, via –clump-kb 500) and linkage disequilibrium (*r*^*2*^ > 0.2, via –clump-r2 0.2). The index SNPs are then taken as independent loci for an outcome of interest^18^.

### Causal inference for ordered multi-categorical exposure

**Figure 1:**
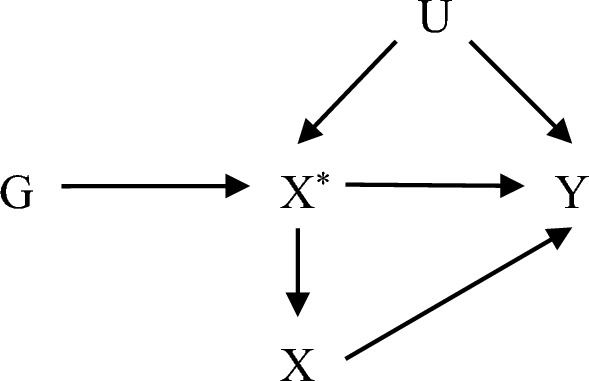
Directed acyclic graph (DAG) of instrumental variables in the causal inference of ordered multi-categorical variables. "G" denotes instrumental variables (genetic variation), "X" indicates exposure, "X^*^" denotes latent variables, "Y" and "U" represent outcome and confounders, respectively.

In order to better fit the CLHLS data while addressing the limitations in the traditional Mendelian randomization, a simple and reliable causal inference method was presented in this study. An ordered multi-categorical variable (exposure) can be regarded as an unknown continuous latent variable divided by the corresponding threshold. Thus, the model for causal inference with the instrumental variables can be expressed as (Fig. 1):

The association between variables can be represented by the following equation:

1 $$ X^{*} = \theta_{0} + \theta G + \gamma U + \varepsilon_{2} $$

2 $$ X = \left[ {\begin{array}{*{20}c} {1,} & {X^{*} < t_{1} } \\ {2,} & {t_{1} < X^{*} < t_{2} } \\ {3,} & {X^{*} > t_{2} } \\ \end{array} } \right. $$

3 $$ Y = \beta_{0} + \beta X^{*} + \alpha U + \varepsilon_{1} $$

$({\text{G}},X,Y)$ are the observable data. Without loss of generality, we assume that $U\sim N(0,{\sigma }_{u}^{2})$, ${\epsilon }_{1}\sim N(0,{\sigma }_{1}^{2})$, ${\epsilon }_{2}\sim N(0,{\sigma }_{2}^{2})$, $G$, $U$, ${\epsilon }_{1}$ and ${\epsilon }_{2}$ are mutual independent of each other. We want to estimate the causal effect ($\beta$) of ${X}^{*}$ on $Y$. Combining the above equation shows that the relationship between $Y$ and $G$ can be expressed by the following equation:

4 $$ Y = \left( {\beta_{0} + \theta_{0} \beta } \right) + \theta \beta G + \left( {\alpha + \gamma \beta } \right)U + \left( {\varepsilon_{1} + \beta \varepsilon_{2} } \right) $$

$G$, $U$, ${\epsilon }_{1}$ and ${\epsilon }_{2}$ are independent of each other, so the parameter $\theta \beta$ can be identified. Regression is performed on G utilizing Y. The regression coefficient is the estimate of the parameter $\theta \beta$. If non-zero $\theta$ can be identified, the parameter $\beta$ can be identified.

Note that

5 $$ P\left( {X = 1{|}G} \right) = P\left( {X^{*} \left\langle {t_{1} } \right|G} \right) = P\left( {\gamma U + \varepsilon_{1} < t_{1} - \theta_{0} - \theta G} \right) $$

6 $$ P\left( {X = 2{|}G} \right) = P\left( {t_{1} < X^{*} \left\langle {t_{2} } \right|G} \right) = P\left( {\gamma U + \varepsilon_{2} < t_{2} - \theta_{0} - \theta G} \right) - P\left( {\gamma U + \varepsilon_{2} < t_{1} - \theta_{0} - \theta G} \right) $$

7 $$ P\left( {X = 3{|}G} \right) = P\left( {X^{*} > t_{2} |G} \right) = P\left( {\gamma U + \varepsilon_{2} > t_{2} - \theta_{0} - \theta G} \right) $$

Since $E\left(\gamma U+{\epsilon }_{2}\right)=0$, $D\left(\gamma U+{\epsilon }_{2}\right)={\gamma }^{2}{\sigma }_{u}^{2}+{\sigma }_{2}^{2}$, let ${\sigma }^{2}={\gamma }^{2}{\sigma }_{u}^{2}+{\sigma }_{2}^{2}$, then

8 $$ P\left( {X = 1{|}G} \right) = {\Phi }\left( {\frac{{t_{1} - \theta_{0} - \theta G}}{\sigma }{ }} \right) $$

9 $$ P\left( {X = 2{|}G} \right) = {\Phi }\left( {\frac{{t_{2} - \theta_{0} - \theta G}}{\sigma }{ }} \right) - {\Phi }\left( {\frac{{t_{1} - \theta_{0} - \theta G}}{\sigma }{ }} \right) $$

10 $$ P\left( {X = 3{|}G} \right) = 1 - {\Phi }\left( {\frac{{t_{2} - \theta_{0} - \theta G}}{\sigma }{ }} \right) $$

where $\Phi$ is the cumulative distribution function of standard normal. From the above equation, it is clear that the parameter $\theta$ is not identifiable. Further we used Eq. (8), which after transformation yields

11 $$ \Phi^{ - 1} \left( {P\left( {X = 1|G} \right)} \right) = \frac{{t_{1} - \theta_{0} }}{\sigma } - G \times \frac{\theta }{\sigma } $$

Subsequently, it is easy to know that $\frac{\theta }{\sigma }$ can be identified by this equation. If one really wants to estimate the causal effect, they could try to estimate ${\sigma }^{2}$ from a separate study. ${\sigma }^{2}$ is connect to heritability of the continuous latent exposure ${X}^{*}$. According to Eq. (1) and the definition of ${\sigma }^{2}$, ${{V}_{{X}^{*}}={V}_{G}+\sigma }^{2}$, where ${V}_{{X}^{*}}$ is the remainder of variance of ${X}^{*}$ after accounting for non-confounding covariants (e.g., age, sex, and first few genotype principal components), and ${V}_{G}$ is variation in ${X}^{*}$ attributed to the genotype. If one knows the heritability ${h}^{2}={V}_{G}/{V}_{{X}^{*}}={V}_{G}/{({V}_{G}+\sigma }^{2})$, one could estimate ${V}_{G}$, then ${\sigma }^{2}$—the part of variation in latent variable ${X}^{*}$ not attributed to genotype could be estimated, and thus one be able to estimate the true effect $\theta$ of the MR/IV model, and lastly, the true effect of ${X}^{*}$ on outcome Y.

For combining all observations, we used the maximum likelihood estimation (which produces smaller variance and mean squared error^19^) for the calculation. The log-likelihood function is given as:

12 $$ log\mathop \prod \limits_{i = 1}^{n} P\left( {X_{i} = x_{i} |G_{i} = g_{i} } \right) = \mathop \sum \limits_{i = 1}^{n} \log \left( {P\left( {X_{i} = x_{i} |G_{i} = g_{i} } \right)} \right) $$

where $P\left({X}_{i}={x}_{i}|{G}_{i}={g}_{i}\right)$ was defined by Eqs. (8–10), which is easily generalizable to exposures of more than 3 levels.

The above derivation still holds if there are multiple instrumental variables (i.e., $G$ is a vector). We can first use the observed data $(G,X)$ to get the estimate $\widehat{\theta }$ of $\theta$ by the maximum likelihood estimation, and then perform a regression on $\widehat{\theta }G$ using $Y$ (same as two-stage regression). The estimated $\widehat{\beta }$ of the causal effect (*β)* can be obtained by this process. Because the estimate of $\theta$ (i.e., the estimate of $\frac{\theta }{\sigma }$) in this method differs from the true value by a constant multiple ($\sigma$), the result of this two-stage estimate also has a difference of a constant multiple ($\sigma$) from the true value. If this estimate is significantly non-zero, then the true causal effect is significantly non-zero. Therefore, this method can be used to infer whether ordered multi-categorical exposures (commonly found in questionnaires) have a significant causal effect on outcome (IVs need to be found with genetic data). In this study, the confidence interval (*CI*) of the causal effect was estimated by the bootstrap (the "boot" package of R software).

We conducted simulations using ten data sets randomly generated (n = 1000, Group A–G in which $Y$ was correlated with ${X}^{*}$ and the last three groups were uncorrelated) combined with repeated sampling (500 times per group), and the results validated the validity and reliability of this method for testing potential causal associations. The simulation results were shown in Table 1.

**Table 1:** Simulation of causal inference for ordered multi-categorical exposure.

Simulation	IV^a^	*β*	$\widehat{\beta }$	95% Confidence interval (*CI*)		
				Normal	Basic	Percentile
Group A	8	1.5	2.503	2.314–2.783	2.315–2.784	2.221–2.690
Group B	6	1.5	2.395	2.067–2.817	2.003–2.781	2.010–2.787
Group C	5	1.5	2.227	1.913–2.577	1.907–2.556	1.898–2.547
Group D	4	2.7	4.419	3.912–5.054	3.848–5.014	3.823–4.990
Group E	6	2.7	4.419	4.022–4.938	4.005–4.916	3.922–4.833
Group F	6	− 2.7	− 4.068	− 4.551–− 3.672	− 4.525–− 3.580	− 4.556–− 3.611
Group G	6	− 1.5	− 2.223	− 2.495–− 2.014	− 2.491–− 1.992	− 2.453–− 1.954
Group H	5	0	− 0.027	− 0.112–0.063	− 0.111–0.064	− 0.118–0.057
Group I	8	0	− 0.008	− 0.073–0.062	− 0.071–0.060	− 0.075–0.056
Group J	3	0	0.048	− 0.067–0.171	− 0.064–0.180	− 0.084–0.160

"a" represents the number of instrumental variables, "Normal" means the *CI* was calculated by the normal approximation method, "Basic" indicates the *CI* was calculated by the basic bootstrap method and "Percentile" stands for the *CI* was calculated by the bootstrap percentage method. The ten data sets were randomly generated with a preset $\sigma$ of 1.5.

Furthermore, the factors ($X$) identified by previous studies as significantly associated with cognitive level ($Y$) were used as exposures and analyzed in conjunction with our causal inference method. The exposures were drinking status, the intake frequency of fish, fruit, garlic, legume, meat, sugar, and vegetable, exercise status, and the participation in activities such as housework, mahjong, open-air activities, pet ownership, reading, and television/radio, respectively.

For the statistically significant results among them, we used the MR-Egger method to test the potential horizontal pleiotropy.

### Role of the funding source

The funder of the study had no role in the study design, data collection, data analysis, data interpretation, or writing of the report. The corresponding authors had full access to all the data in the study and had final responsibility for the decision to submit for publication.

### Ethics approval and consent to participate

The Research Ethics Committees of Peking University (number IRB00001052-13074) and Duke University granted approval for the CLHLS, including collections of questionnaire data with written informed consent before participation. The study was performed in accordance with the Declaration of Helsinki. This study only showed the secondary aggregated data, and we did not include any data of their personal information, including name, identity information, address, telephone number, etc. None of the authors in this study had access to identifying patient information during the analysis of the data.

## Results

**Figure 2:**
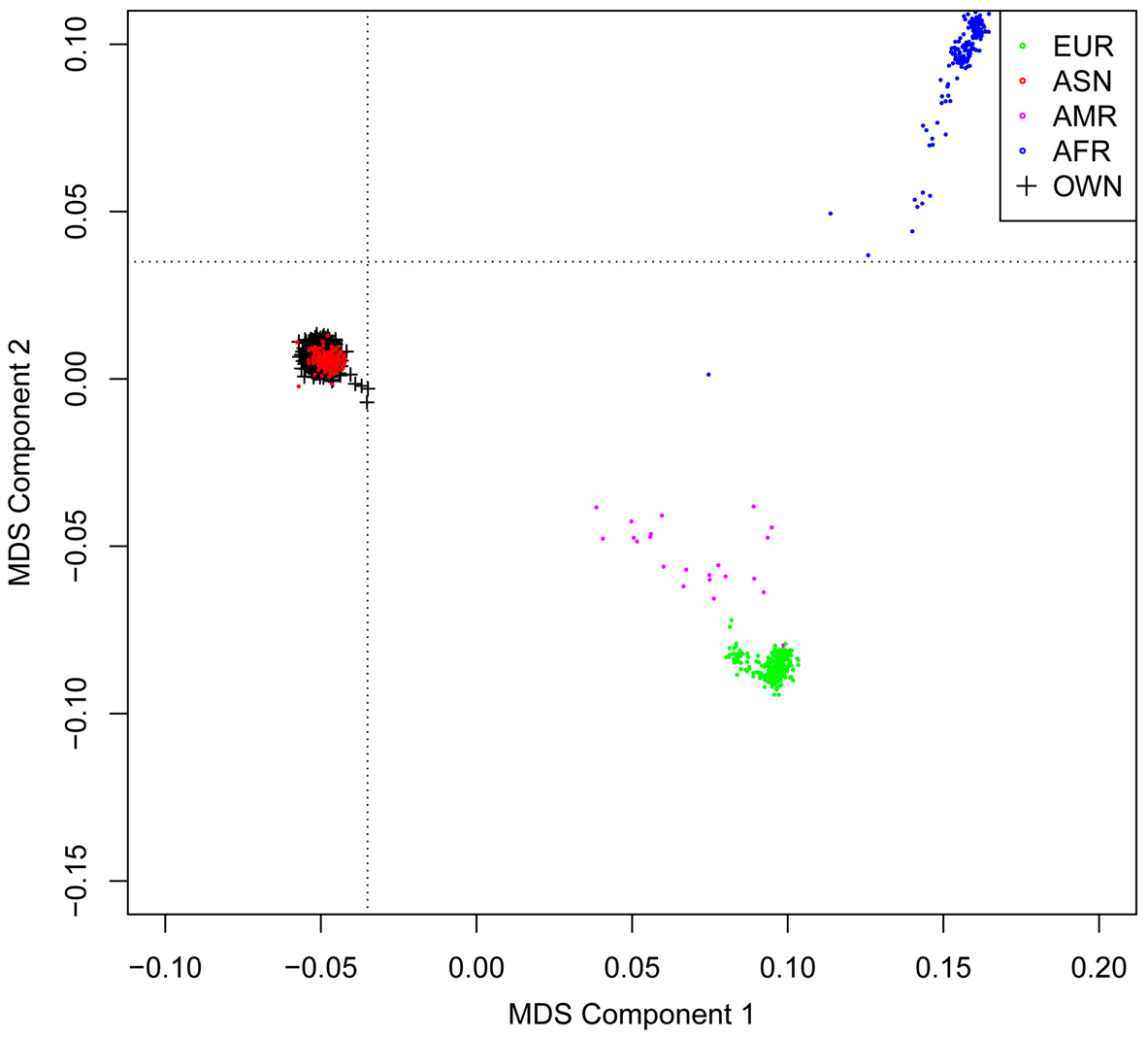
Population stratification of the sample (multidimensional scaling method). "EUR" stands for European, "ASN" for Asian, "AMR" for American, and "AFR" represents African origin (this part of the data was from the 1000 Genomes Project). "OWN" represents the data involved in this study.

After quality control, a total of 897 older adults were included in the study. Their mean (SD) age was at 97.19 (6.70) years and their mean (SD) cognitive score was 21.42 (7.34). The genetic data covered 3 240 266 loci (autosomes 1–22). Meanwhile, the population distribution of our sample was highly homologous to the Asian population in the 1000 Genomes Project, and there was no population stratification in this study. The results were shown in Fig. 2.

### Results of genome-wide association analysis

Genome-wide association analysis detected 512 significant loci with *p* < 1 × 10^–5^ adjusted for covariates. Among them, four independent loci were associated with cognition. Seventy-eight independent loci were detected for categorical exposures, distributed among drinking status (6), fish (7), fruit (3), garlic (5), legume (8), meat (8), sugar (2), vegetable (4), exercise status (7), housework (6), mahjong (3), open-air activities (5), pet ownership (6) read (4), and TV/radio (4). In addition, 11 and 3 independent loci were found in education and stroke/CVD, respectively. The results were shown in Additional file 2.

Among the above loci, one locus reached genome-wide significance (*p* < 5 × 10^–8^)—rs78069066 (*p* = 1.21 × 10^–8^, from drinking status). The Manhattan and Q-Q plots of GWAS results for each variable were detailed in Additional file 3.

### Causal inference results for ordered multi-categorical exposures

We explored potential causal associations using a causal inference approach for ordered multi-categorical exposures. Instrumental variables were selected from independent loci that were significant in GWAS, and loci with known confounding were excluded. Finally, a total of 78 independent SNPs were selected as instrumental variables. The IVs included for each exposure were the same as the independent loci screened above, as detailed in additional file 2.

After multiple replicate calculations (500 replicates for each exposure), the results showed a positive causal association between individuals' exercise status and their own cognitive level ($\widehat{\beta }$ = 1.883, 95%*CI* 0.182—3.512). In contrast, there was no causal association between diet, activity, and cognitive level of older adults. The specific results were shown in Table 2.

**Table 2:** Causal inference results for ordered multi-categorical exposure.

Variable	IV^a^	n^b^	$\widehat{\beta }$	95% *Cl* (Basic)	*p*^c^
Drink	6	866	− 1.127	− 2.689–0.285	0.138
Diet*					
Fish*	7	882	− 0.752	− 2.551–1.151	0.426
Fruit*	3	874	− 0.835	− 3.427–1.612	0.516
Garlic*	5	877	0.710	− 0.885–2.601	0.425
Legume*	8	858	0.830	− 1.501–2.671	0.435
Meat*	8	827	− 0.164	− 2.299–1.835	0.877
Sugar*	2	892	1.126	− 1.577–3.488	0.384
Vegetable*	4	869	0.291	− 1.523–2.425	0.773
Exercise status	7	855	1.883	0.182–3.512	0.027
Activity*					
Housework*	6	884	− 1.282	− 3.676–1.175	0.300
Mahjong*	3	879	− 1.075	− 2.431–0.002	0.083
Open-air*	5	877	− 1.438	− 3.844–0.703	0.215
Pet ownership*	6	862	− 1.080	− 2.192–0.064	0.061
Read*	4	876	− 0.516	− 2.775–1.450	0.636
TV/radio*	4	885	− 0.251	− 2.435–1.726	0.813

“*” represents that the variable was reverse coded, with higher values indicating lower frequency, as described in detail in Additional file 1. "a" represents the number of instrumental variables, "b" represents the number of samples for each exposure (excluding individuals with missing information on the corresponding IVs), and "c" indicates that the *p* value is reverse derived from *CI* through a normal distribution.

In response to the statistically significant results, a further sensitivity analysis confirmed that there was no horizontal pleiotropy between cognitive level and exercise status (*p* > 0.05, Table 3). This proved that the causal effect described above was valid and reliable.

**Table 3:** Results of the horizontal pleiotropy test.

Variable	Egger intercept	S.E	*p* value
Exercise status	1.203	1.221	0.370

## Discussion

Currently, Mendelian randomization is the mostly commonly accepted method of causal inference in medical field. The traditional MR, however, has a relatively limited field of application and usually prefers causal inference of exposure and outcome in the form of continuous variables. If categorical variables were directly analyzed as exposures, the estimated causal effects would be inaccurate^10,20^. Although the methodology in this field has been expanded somewhat in recent years, causal inference involving categorical variables has focused more on cases where the outcome is a categorical variable^21–23^, and studies of categorical exposures are still lacking.

Causal inference with exposure in the form of categorical variables such as binary categories was highly sensitive to the choice of thresholds. Since categorical exposures typically only contain effects associated with boundary points and category changes and do not adequately capture subtle changes (failure to change category) in its original variables (continuous exposures), true causal effect are often difficult to explore. In addition, the causal estimates calculated by MR can sometimes be difficult to interpret. Therefore, it has been suggested that MR should focus more on testing for potential causal effects rather than trying to compute estimates of causal effects^24,25^.

The innovative method in this study treats ordered multi-categorical variables as divided from continuous variables by specific boundary points, and on this basis, the causal association between ordered multi-categorical exposures and continuous outcomes was inferred and uncovered through maximum likelihood estimation. This method can easily and effectively explore the causal associations between exposures and outcomes. Meanwhile, this added a new theoretical basis to the field and provided evidence to support the development of subsequent methods. In addition, this method estimated causal effects that differed from the true effects by a constant multiple. This situation was consistent with the idea mentioned in previous studies that using coarsened measures (categorical exposures) as exposures for estimation leads to bias, which would amplify or reduce their effect estimates without inverting their sign^10^.

In addition, the study confirmed a potential causal association between exercise status and individual cognitive level through our method. The results suggested that exercise could delay the cognitive decline. A recent international study that analyzed the brains of hundreds of deceased older adults found that individuals who participated more in daily exercise had higher levels of presynaptic proteins and better synaptic integrity in their brains during old age^26^ This is the first time that the positive effects of exercise on synaptic function have been demonstrated in humans. Earlier studies have shown that only 10 min of moderate intensity running per day can improve mood and cognitive performance^27^. Exercise such as running increases blood flow to five cortical areas: l-DLPFC, l-FPA, r-DLPFC, r-VLPF and r-FPA. The stimulation received by these areas played a very important role in improving mood and cognition. Regardless of the activity, high-frequency exercise invariably increases the cognitive load of older adults. After exercise with high cognitive load, it enhanced functional connectivity in the superior frontal gyrus and prefrontal cortex and reduced functional connectivity in the middle occipital gyrus and postcentral gyrus at rest, which in turn delayed cognitive decline^28^.

On the other hand, the positive effects from exercise were not really lasting^26^. This also demonstrated to some extent the importance of maintaining a long-term, healthy exercise habit in the older adults. The adoption of measures such as appropriate physical activity was necessary no matter what age. As Dr. Robert S. Wilson said^29^, "It's never too late to start participating in such activities. Even in your 80 s, cognitive stimulation activities can delay the onset of Alzheimer's disease.

## Conclusion

The causal inference method developed in this study combined with the maximum likelihood estimation addressed the problem of difficult causal inference for ordered multi-categorical exposures and compensated for the limitations of traditional MR methods. This not only provides us a practical tool, but also a different way of developing methodologies in this field. In addition, we uncovered a potential positive causal association between exercise and cognition through a more in-depth analysis based on previous studies. This result further confirmed the positive effect of exercise on cognitive health in Chinese older adults and added stronger evidence for delaying cognitive decline in the older individuals.

### Supplementary Information

Supplementary Information 1.

Supplementary Information 2.

Supplementary Figures.

## Data Availability

The datasets generated and/or analyzed during the current study are available in the Peking University Open Research Data repository. The code can be obtained by contacting the corresponding author. [https://opendata.pku.edu.cn/dataset.xhtml?persistentId=doi%3A10.18170%2FDVN%2FWBO7LK].
