# Supplementary material for: Exercise may delay cognitive decline in Chinese older adults: a causal inference for ordered multi-categorical exposures with a Mendelian randomization approach
Source: Sci Rep. 2024 Jun 6;14:13007. doi: 10.1038/s41598-024-59326-7 (PMC11156672; doi:10.1038/s41598-024-59326-7)
Supplement: Supplementary file 1 — Supplementary Information 1. [file 41598_2024_59326_MOESM1_ESM.docx]

| **List of variables explanation** | |
| --- | --- |
| **Variables** | **Explanation** |
| Activity-housework* | The values of the activity component variables were categorized into three levels, representing the frequency of participation. Values from low to high represent "frequently", "occasionally" and "rarely or never". |
| Activity-mahjong* |  |
| Activity-open-air* |  |
| Activity-pet ownership* |  |
| Activity-read* |  |
| Activity-TV/radio* |  |
| Diet-fish* | The values of the diet component variables were categorized into three levels, representing the frequency of food intake. Values from low to high represent "frequently", "occasionally" and "rarely or never". |
| Diet-fruit* |  |
| Diet-garlic* |  |
| Diet-legume* |  |
| Diet-meat* |  |
| Diet-sugar* |  |
| Diet-vegetable* |  |
| Drinking status* | "1"-never drinking; "2"-long-term drinking and abstinent |
| Education | The variables represented the number of years of education and were recorded in years. |
| Exercise status* | "1"-never exercise; "2"-exercise |
| Stroke/CVD | Participants self-reported whether they were (had been) sick: "1" - sick; "2" - not sick; "3" - not sure |
| Note: "*" represents the variable was included in the study as an exposure | |
