# Supplementary material for: Exercise may delay cognitive decline in Chinese older adults: a causal inference for ordered multi-categorical exposures with a Mendelian randomization approach
Source: Sci Rep. 2024 Jun 6;14:13007. doi: 10.1038/s41598-024-59326-7 (PMC11156672; doi:10.1038/s41598-024-59326-7)
Supplement: Supplementary file 2 — Supplementary Information 2. [file 41598_2024_59326_MOESM2_ESM.docx]

| **Independent loci screening results** | | | | | | | | | |
| --- | --- | --- | --- | --- | --- | --- | --- | --- | --- |
| Variable | CHR | SNP | A1/A2 | BP | *β* / *OR* (95%*CI*) ^a^ | S.E. | *p*-value | KB | Gene / Scope (intersects with aggregation area) |
| Cognition | 1 | rs10907306 | A/T | 18699951 | -1.759 (-2.458 - -1.061 ) | 0.356 | 9.52E-07 | 9.964 | IGSF21 |
|  | 1 | rs1633312 | A/G | 158955151 | -2.231 (-3.132 - -1.330 ) | 0.460 | 1.43E-06 | 13.925 | TMEM181 (2.32kb downstream) |
|  | 8 | rs12550397 | A/G | 69901097 | 2.151 (1.247 - 3.056 ) | 0.462 | 3.64E-06 | 0.491 | LOC100505718 |
|  | 3 | rs1474272 | C/T | 71254193 | -2.078 (-2.973 - -1.183 ) | 0.457 | 6.05E-06 | 0.001 | FOXP1 |
| Drinking status | 2 | rs1560262 | A/G | 41808308 | 1.893 (1.438 - 2.493 ) | 0.140 | 5.44E-06 | 0.001 | RPAP1 (1.66kb downstream) |
|  | 5 | rs11745163 | A/G | 136482547 | 1.862 (1.417 - 2.446 ) | 0.139 | 8.01E-06 | 0.001 | SPOCK1 |
|  | 7 | rs60024014 | G/A | 15954602 | 2.293 (1.630 - 3.226 ) | 0.174 | 1.88E-06 | 51.043 | SAMSN1-AS1 (0.8kb downstream) |
|  | 12 | rs78069066 | A/G | 112337924 | 2.891 (2.007 - 4.165 ) | 0.186 | 1.21E-08 | 0.001 | ADAM1A |
|  | 12 | rs12229654 | G/T | 111414461 | 2.712 (1.839 - 3.998 ) | 0.198 | 4.76E-07 | 85.235 | CCDC63,LOC100131138,MYL2 |
|  | 17 | rs2945393 | G/A | 25883037 | 2.059 (1.555 - 2.726 ) | 0.143 | 4.59E-07 | 41.560 | KSR1 |
| Diet |  |  |  |  |  |  |  |  |  |
| Fish | 1 | rs74575830 | A/G | 191184679 | 2.267 (1.583 - 3.248 ) | 0.183 | 8.08E-06 | 6.472 | HIBCH (0.92kb upstream) |
|  | 5 | rs1510408 | A/C | 84182336 | 1.790 (1.385 - 2.312 ) | 0.131 | 8.36E-06 | 0.001 | COQ2 (2.64kb downstream) |
|  | 8 | rs67971905 | C/A | 14409687 | 1.700 (1.348 - 2.142 ) | 0.118 | 7.14E-06 | 0.001 | SGCZ |
|  | 11 | rs77900940 | G/A | 127079398 | 1.808 (1.401 - 2.333 ) | 0.130 | 5.26E-06 | 1.383 | LOC11927123 (3.12kb upstream) |
|  | 13 | rs912160 | T/C | 45319586 | 1.756 (1.369 - 2.252 ) | 0.127 | 9.13E-06 | 0.001 | TP53RK (1.31kb upstream) |
|  | 15 | rs7165083 | G/A | 66872614 | 2.200 (1.551 - 3.119 ) | 0.178 | 9.71E-06 | 0.001 | LINC1169 (1.91kb downstream) |
|  | 16 | rs11076161 | A/G | 56673148 | 1.802 (1.401 - 2.317 ) | 0.128 | 4.51E-06 | 0.006 | MT1A |
| Fruit | 1 | rs6656785 | A/G | 75005776 | 0.524 (0.406 - 0.678 ) | 0.131 | 8.13E-07 | 6.862 | FPGTTNNI3K，TNNI3K |
|  | 1 | rs6669244 | C/T | 20834789 | 1.972 (1.464 - 2.657 ) | 0.152 | 7.83E-06 | 18.950 | MUL1 |
|  | 2 | rs9309516 | T/C | 77727037 | 2.155 (1.573 - 2.955 ) | 0.161 | 1.81E-06 | 32.262 | LRRTM4 |
| Garlic | 2 | rs7601465 | G/A | 77339715 | 0.584 (0.461 - 0.739 ) | 0.120 | 7.84E-06 | 0.001 | LRRTM4 |
|  | 5 | rs12659780 | T/C | 102064637 | 1.725 (1.357 - 2.193 ) | 0.123 | 8.62E-06 | 0.001 | FLJ42969 (0.36kb downstream) |
|  | 7 | rs10237897 | A/G | 40938720 | 2.111 (1.519 - 2.932 ) | 0.168 | 8.50E-06 | 0.001 | ZFP69 (4.58kb downstream) |
|  | 11 | rs4121639 | T/G | 114241636 | 1.625 (1.315 - 2.007 ) | 0.108 | 6.76E-06 | 0.001 | FLJ3453 (1.17kb upstream) |
|  | 19 | rs10416140 | C/T | 9862634 | 2.426 (1.680 - 3.504 ) | 0.188 | 2.28E-06 | 197.189 | C19orf82，FBXL12，ZNF561，ZNF562，ZNF812，ZNF846 |
| Legume | 3 | rs9853046 | G/A | 130540562 | 2.076 (1.525 - 2.826 ) | 0.157 | 3.51E-06 | 0.001 | SH2D3C (0.49kb upstream) |
|  | 3 | rs12635513 | G/A | 82635918 | 2.164 (1.541 - 3.040 ) | 0.173 | 8.41E-06 | 68.836 | ZFAND1 (2.38kb upstream) |
|  | 4 | rs74873220 | A/G | 10083872 | 2.931 (1.844 - 4.658 ) | 0.236 | 5.37E-06 | 3.175 | WDR1 |
|  | 4 | rs706309 | C/T | 140478478 | 2.319 (1.608 - 3.345 ) | 0.187 | 6.67E-06 | 0.001 | SETD7 (0.91kb upstream) |
|  | 6 | rs971927 | T/C | 124876943 | 2.063 (1.524 - 2.794 ) | 0.155 | 2.83E-06 | 0.001 | NKAIN2 |
|  | 11 | rs745870 | G/T | 76027936 | 0.494 (0.362 - 0.675 ) | 0.159 | 9.32E-06 | 0.001 | ZP3 (1.96kb downstream) |
|  | 12 | rs4761794 | C/G | 52819181 | 2.879 (1.811 - 4.575 ) | 0.236 | 7.70E-06 | 3.525 | KRT75 |
|  | 15 | rs67839313 | C/T | 40619724 | 2.248 (1.613 - 3.134 ) | 0.169 | 1.72E-06 | 0.837 | LRRK2 (0.91kb downstream) |
| Meat | 2 | rs6741930 | A/G | 172396366 | 2.670 (1.755 - 4.062 ) | 0.214 | 4.48E-06 | 1.946 | CYBRD1 |
|  | 3 | rs767460 | G/A | 2741787 | 2.740 (1.826 - 4.111 ) | 0.207 | 1.12E-06 | 11.339 | CNTN4 |
|  | 4 | rs74762973 | A/G | 150713843 | 2.155 (1.548 - 3.000 ) | 0.169 | 5.36E-06 | 138.940 | NOS3 (2.16kb upstream) |
|  | 9 | rs1414202 | C/T | 11359656 | 2.540 (1.693 - 3.812 ) | 0.207 | 6.71E-06 | 49.516 | TNP2 (2.57kb downstream) |
|  | 11 | rs111256489 | A/G | 60769819 | 2.892 (1.877 - 4.457 ) | 0.221 | 1.47E-06 | 16.656 | CD6 |
|  | 17 | rs112537719 | T/C | 5482389 | 3.251 (1.933 - 5.466 ) | 0.265 | 8.74E-06 | 44.547 | NLRP1 |
|  | 18 | rs2315175 | T/C | 39198804 | 2.098 (1.527 - 2.883 ) | 0.162 | 4.93E-06 | 0.001 | KRTAP1-1 (1.91kb upstream) |
|  | 21 | rs2251990 | G/A | 32834757 | 2.138 (1.552 - 2.947 ) | 0.164 | 3.43E-06 | 3.779 | TIAM1 |
| Sugar | 3 | rs7613462 | G/A | 125811541 | 2.027 (1.487 - 2.761 ) | 0.158 | 7.65E-06 | 15.042 | ALDH1L1，ALDH1L1AS1，SLC41A3 |
|  | 5 | rs10072153 | C/G | 100302331 | 0.576 (0.460 - 0.722 ) | 0.115 | 1.63E-06 | 0.595 | POP7 (1.34kb downstream) |
| Vegetable | 2 | rs7583479 | T/C | 10473779 | 5.242 (2.788 - 9.859 ) | 0.322 | 2.73E-07 | 0.001 | HPCAL1 |
|  | 13 | rs4942709 | G/T | 48447563 | 4.011 (2.180 - 7.381 ) | 0.311 | 8.06E-06 | 4.322 | SNAR-A12 (0.62kb downstream) |
|  | 15 | rs28601610 | G/A | 93851751 | 3.507 (2.050 - 6.000 ) | 0.274 | 4.68E-06 | 0.001 | HEPHL1 (4.38kb upstream) |
|  | 16 | rs11646848 | G/A | 88635310 | 3.690 (2.114 - 6.440 ) | 0.284 | 4.34E-06 | 25.001 | ZC3H18 |
| Exercise status | 1 | rs12041851 | A/G | 101767274 | 2.024 (1.510 - 2.712 ) | 0.149 | 2.34E-06 | 5.157 | TBC1D8 (0.57kb upstream) |
|  | 3 | rs2197328 | C/A | 4180589 | 0.582 (0.463 - 0.733 ) | 0.117 | 3.89E-06 | 9.701 | SIRT6 (2.7kb upstream) |
|  | 6 | rs4708389 | A/G | 169297420 | 0.603 (0.484 - 0.751 ) | 0.112 | 6.16E-06 | 0.001 | FAM196B (6.72kb downstream) |
|  | 10 | rs703970 | A/C | 80953136 | 1.702 (1.345 - 2.153 ) | 0.120 | 9.24E-06 | 0.001 | ZMIZ1 |
|  | 11 | rs7115731 | C/A | 87531398 | 0.483 (0.351 - 0.663 ) | 0.162 | 7.06E-06 | 14.608 | LOC11928737 (3.66kb downstream) |
|  | 13 | rs9557573 | T/C | 101690164 | 0.578 (0.465 - 0.719 ) | 0.111 | 8.63E-07 | 1.858 | NALCNAS1 |
|  | 20 | rs3844459 | A/G | 6152452 | 0.507 (0.376 - 0.683 ) | 0.153 | 8.30E-06 | 0.001 | NLGN4X (5.53kb upstream) |
| Activity |  |  |  |  |  |  |  |  |  |
| Housework | 1 | rs6696698 | G/A | 91658368 | 2.048 (1.524 - 2.752 ) | 0.151 | 1.96E-06 | 22.193 | TMEM64 (0.24kb upstream) |
|  | 3 | rs12486359 | C/G | 28020989 | 1.802 (1.415 - 2.295 ) | 0.123 | 1.79E-06 | 0.588 | MTIF3 (3.75kb upstream) |
|  | 4 | rs17045384 | T/C | 113951743 | 0.558 (0.436 - 0.712 ) | 0.125 | 2.97E-06 | 0.001 | ANK2 |
|  | 6 | rs11756310 | T/C | 91724888 | 2.945 (1.841 - 4.710 ) | 0.240 | 6.55E-06 | 0.001 | HFM1 (1.43kb downstream) |
|  | 15 | rs4932439 | G/A | 89401109 | 1.759 (1.391 - 2.224 ) | 0.120 | 2.41E-06 | 0.001 | ACAN |
|  | 20 | rs2021349 | G/A | 6181948 | 1.879 (1.444 - 2.445 ) | 0.134 | 2.70E-06 | 0.001 | PFKFB3 (4.89kb downstream) |
| Mahjong | 10 | rs3121490 | C/T | 115420776 | 0.446 (0.317 - 0.627 ) | 0.174 | 3.52E-06 | 8.257 | NRAP |
|  | 14 | rs7156078 | G/A | 66238701 | 0.468 (0.339 - 0.646 ) | 0.165 | 4.08E-06 | 48.888 | FUT8 |
|  | 15 | rs3743024 | G/A | 42118971 | 0.405 (0.274 - 0.601 ) | 0.201 | 6.93E-06 | 2.568 | JMJD7，JMJD7PLA2G4B，MAPKBP1 |
| Open-air | 1 | rs61828616 | T/C | 230817480 | 2.201 (1.566 - 3.093 ) | 0.174 | 5.57E-06 | 4.005 | COG2 |
|  | 8 | rs12542480 | A/C | 69457220 | 1.590 (1.297 - 1.949 ) | 0.104 | 8.04E-06 | 0.001 | C8orf34 |
|  | 10 | rs10906681 | G/A | 14493853 | 1.728 (1.361 - 2.192 ) | 0.122 | 6.81E-06 | 0.001 | CD97 (1.9kb downstream) |
|  | 11 | rs10791725 | G/A | 104585757 | 0.615 (0.496 - 0.763 ) | 0.110 | 9.40E-06 | 0.001 | MIR23A (1.96kb upstream) |
|  | 16 | rs429790 | C/A | 84471642 | 0.608 (0.494 - 0.749 ) | 0.106 | 2.86E-06 | 0.001 | ATP2C2 |
| Pet ownership | 3 | rs2324494 | A/G | 76309408 | 0.537 (0.414 - 0.698 ) | 0.134 | 3.29E-06 | 0.001 | CNTNAP4 (1.77kb downstream) |
|  | 5 | rs66608099 | A/G | 90356564 | 0.467 (0.343 - 0.636 ) | 0.158 | 1.39E-06 | 13.375 | GPR98 |
|  | 7 | rs74388387 | C/A | 78666643 | 0.482 (0.361 - 0.644 ) | 0.148 | 7.54E-07 | 0.001 | MAGI2 |
|  | 12 | rs78033844 | A/G | 59368925 | 0.314 (0.193 - 0.510 ) | 0.248 | 2.93E-06 | 0.440 | LINC1135 (3.54kb upstream) |
|  | 18 | rs595499 | C/G | 11999724 | 0.538 (0.416 - 0.696 ) | 0.131 | 2.34E-06 | 1.122 | IMPA2 |
|  | 20 | rs11696185 | T/C | 52366310 | 0.517 (0.388 - 0.689 ) | 0.147 | 7.04E-06 | 1.675 | TRAM2 (4.11kb downstream) |
| Read | 1 | rs11584105 | T/C | 58827683 | 0.410 (0.281 - 0.598 ) | 0.192 | 3.65E-06 | 29.317 | LOC283194 (1.76kb upstream) |
|  | 4 | rs4696891 | T/C | 8654825 | 0.415 (0.283 - 0.609 ) | 0.196 | 6.83E-06 | 0.001 | HULC (0.75kb upstream) |
|  | 13 | rs17075652 | T/C | 31781412 | 0.231 (0.123 - 0.433 ) | 0.321 | 4.94E-06 | 0.001 | B3GALTL |
|  | 14 | rs17544968 | T/C | 81457303 | 0.395 (0.266 - 0.588 ) | 0.203 | 4.72E-06 | 17.842 | TSHR |
| TV/radio | 1 | rs227099 | A/G | 70134530 | 1.609 (1.324 - 1.955 ) | 0.100 | 1.78E-06 | 28.516 | LINC593 (0.78kb upstream) |
|  | 5 | rs13153619 | T/G | 147723506 | 0.626 (0.512 - 0.765 ) | 0.102 | 4.79E-06 | 58.090 | LOC102546294，SPINK7，SPINK9 |
|  | 10 | rs2421620 | G/A | 93399313 | 1.703 (1.375 - 2.108 ) | 0.109 | 1.05E-06 | 34.876 | LOC643339 (1.78kb downstream) |
|  | 11 | rs1923295 | T/C | 35442608 | 1.591 (1.304 - 1.940 ) | 0.101 | 4.65E-06 | 0.001 | ACACA (0.68kb downstream) |
| Education | 1 | rs4660770 | C/T | 44541996 | 0.556 (0.314 - 0.798 ) | 0.124 | 7.66E-06 | 0.001 | TCEB3CL (0.73kb downstream) |
|  | 2 | rs76708726 | G/A | 154343387 | 1.099 (0.629 - 1.569 ) | 0.24 | 5.25E-06 | 0.114 | MRPL22 (5.58kb upstream) |
|  | 2 | rs116878817 | C/T | 220071039 | 0.678 (0.385 - 0.972 ) | 0.15 | 6.64E-06 | 0.001 | ZFAND2B (0.47kb downstream) |
|  | 6 | rs9363962 | T/G | 69400984 | 0.783 (0.450 - 1.116 ) | 0.17 | 4.66E-06 | 0.001 | BAI3 |
|  | 8 | rs8180896 | C/T | 85990757 | 1.256 (0.735 - 1.777 ) | 0.266 | 2.68E-06 | 57.043 | LRRCC1 |
|  | 8 | rs11775034 | A/G | 6894163 | 0.571 (0.334 - 0.809 ) | 0.121 | 2.85E-06 | 0.001 | OR1A2 (2.27kb upstream) |
|  | 8 | rs10093742 | A/G | 24136679 | 0.847 (0.479 - 1.216 ) | 0.188 | 7.48E-06 | 0.001 | LINC691 (4.79kb downstream) |
|  | 9 | rs12000633 | A/G | 114747481 | 0.835 (0.477 - 1.193 ) | 0.183 | 5.50E-06 | 0.001 | RASA3 (0.29kb downstream) |
|  | 12 | rs71456010 | A/G | 71400552 | 0.741 (0.419 - 1.063 ) | 0.164 | 7.51E-06 | 0.001 | PIN4 (0.97kb downstream) |
|  | 16 | rs4125033 | A/G | 10646459 | 0.559 (0.322 - 0.797 ) | 0.121 | 4.50E-06 | 1.049 | EMP2 |
|  | 22 | rs5762032 | C/T | 27622115 | 0.640 (0.377 - 0.903 ) | 0.134 | 2.15E-06 | 6.614 | NUFIP2 (0.95kb upstream) |
| Strok/CVD | 3 | rs1993904 | C/G | 141003354 | 3.908 (2.135 - 7.153 ) | 0.309 | 9.95E-06 | 0.001 | PXYLP1 |
|  | 15 | rs79017233 | T/C | 70237704 | 5.090 (2.614 - 9.913 ) | 0.34 | 1.71E-06 | 16.287 | SRSF5 (1.18kb upstream) |
|  | 18 | rs11663076 | T/A | 29948616 | 4.060 (2.274 - 7.249 ) | 0.296 | 2.17E-06 | 5.612 | GAREM |
| Note: "upstream" and "downstream" represent the closest upstream or downstream genes to the site, respectively. The *β* at "a" represent the result for cognition (continuous variable), and the results for other categorical variables correspond to *OR*. | | | | | | | | | |
