# Supplementary material for: Exercise may delay cognitive decline in Chinese older adults: a causal inference for ordered multi-categorical exposures with a Mendelian randomization approach
Source: Sci Rep. 2024 Jun 6;14:13007. doi: 10.1038/s41598-024-59326-7 (PMC11156672; doi:10.1038/s41598-024-59326-7)
Supplement: Supplementary file 3 — Supplementary Figures. [file 41598_2024_59326_MOESM3_ESM.docx]

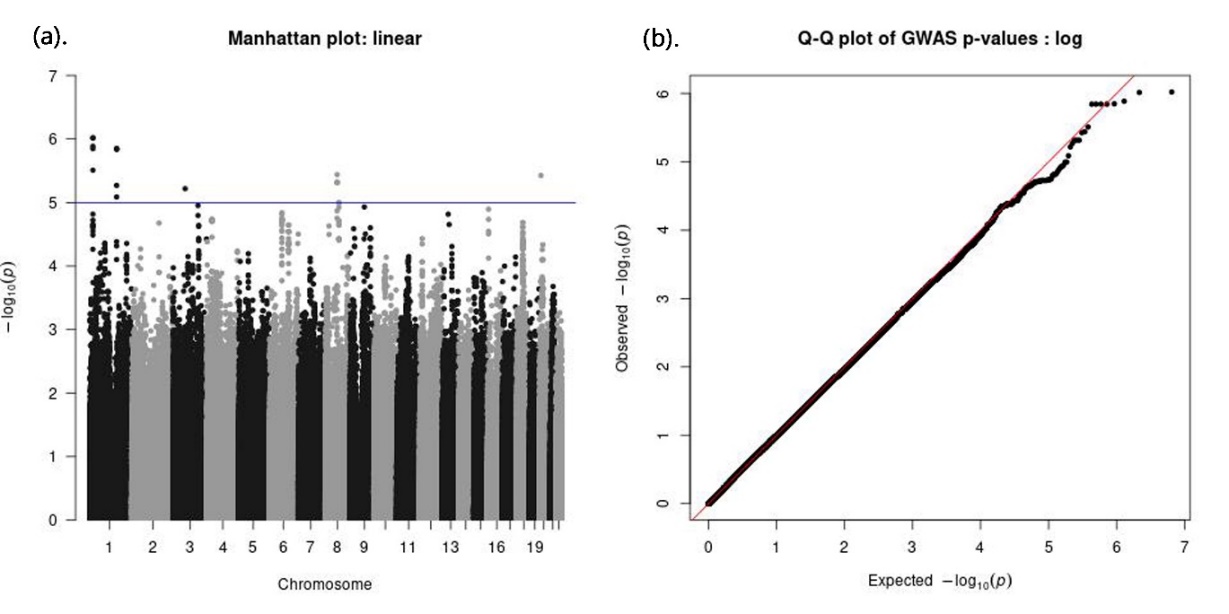


Figure.S1 Manhattan plot and Q-Q plot for cognition (GIF = 1.011)


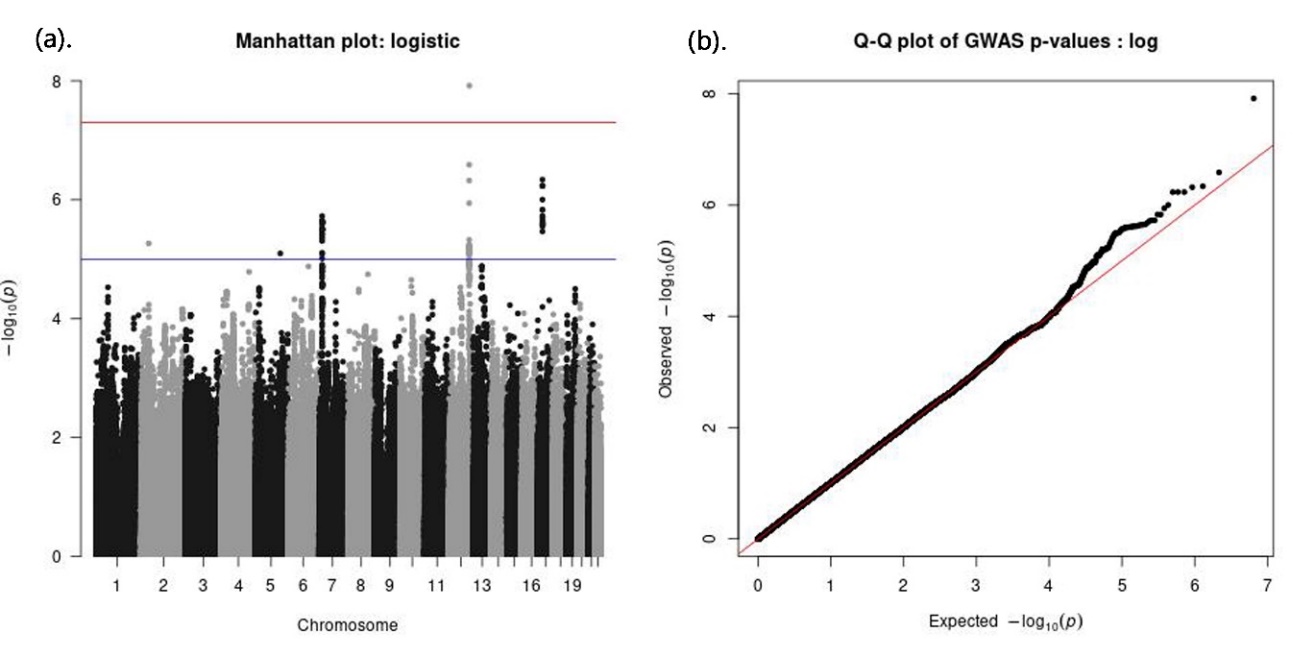


Figure.S2 Manhattan plot and Q-Q plot for drinking status (GIF = 1.019)


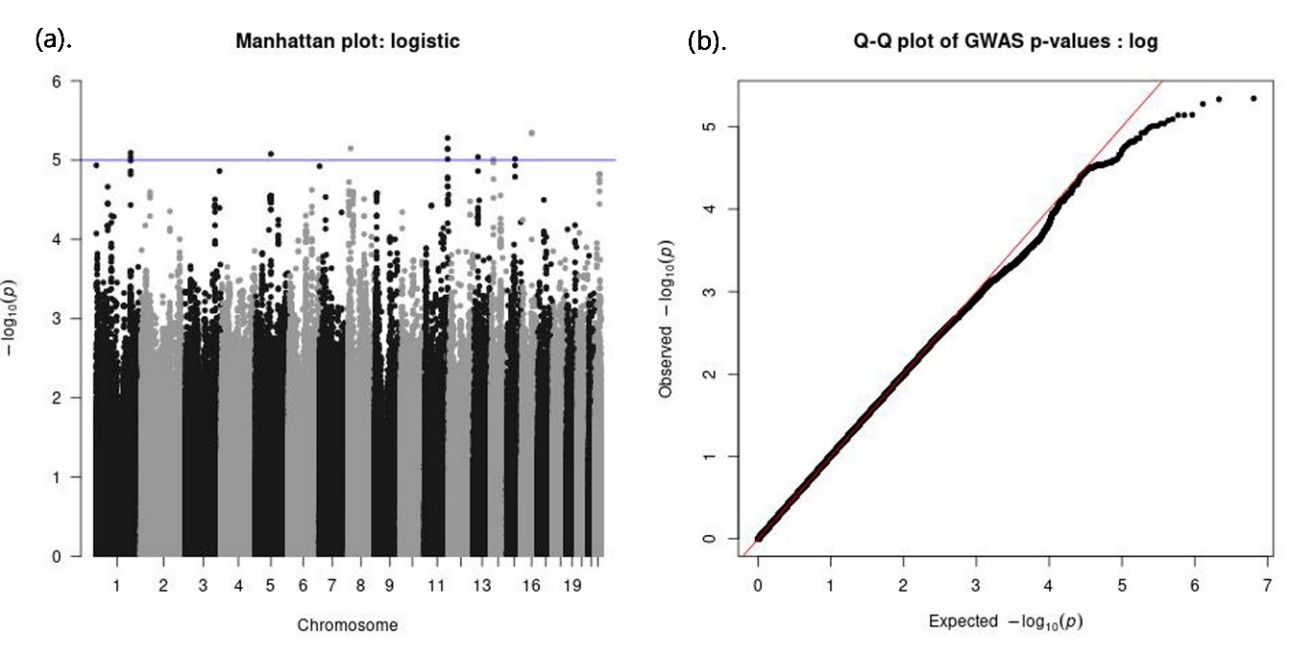


Figure.S3 Manhattan plot and Q-Q plot for fish intake frequency (GIF = 1.023)


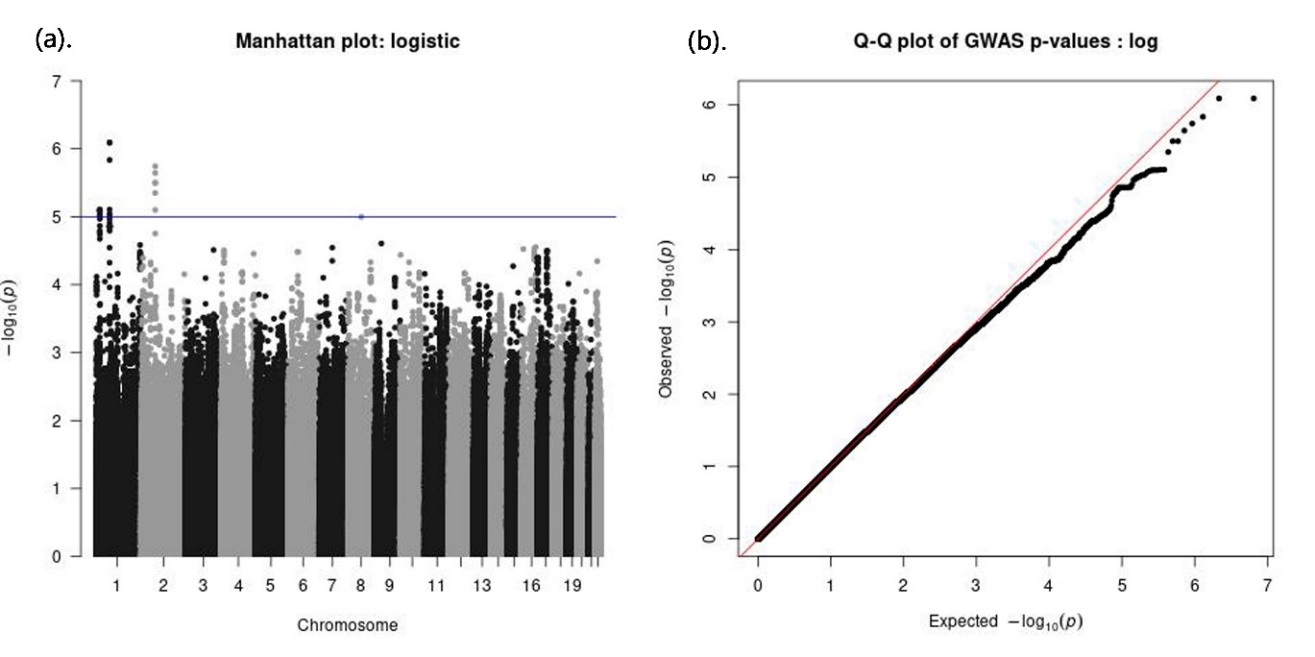


Figure.S4 Manhattan plot and Q-Q plot for fruit intake frequency (GIF = 1.008)


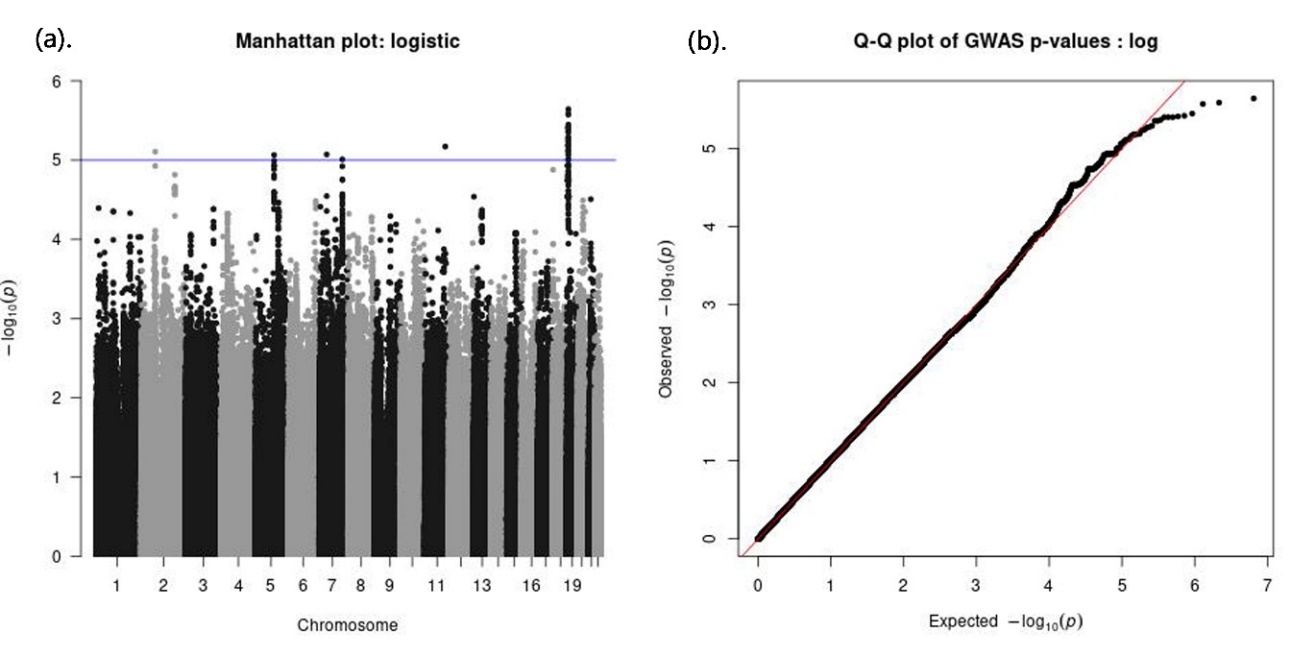


Figure.S5 Manhattan plot and Q-Q plot for garlic intake frequency (GIF = 1.020)


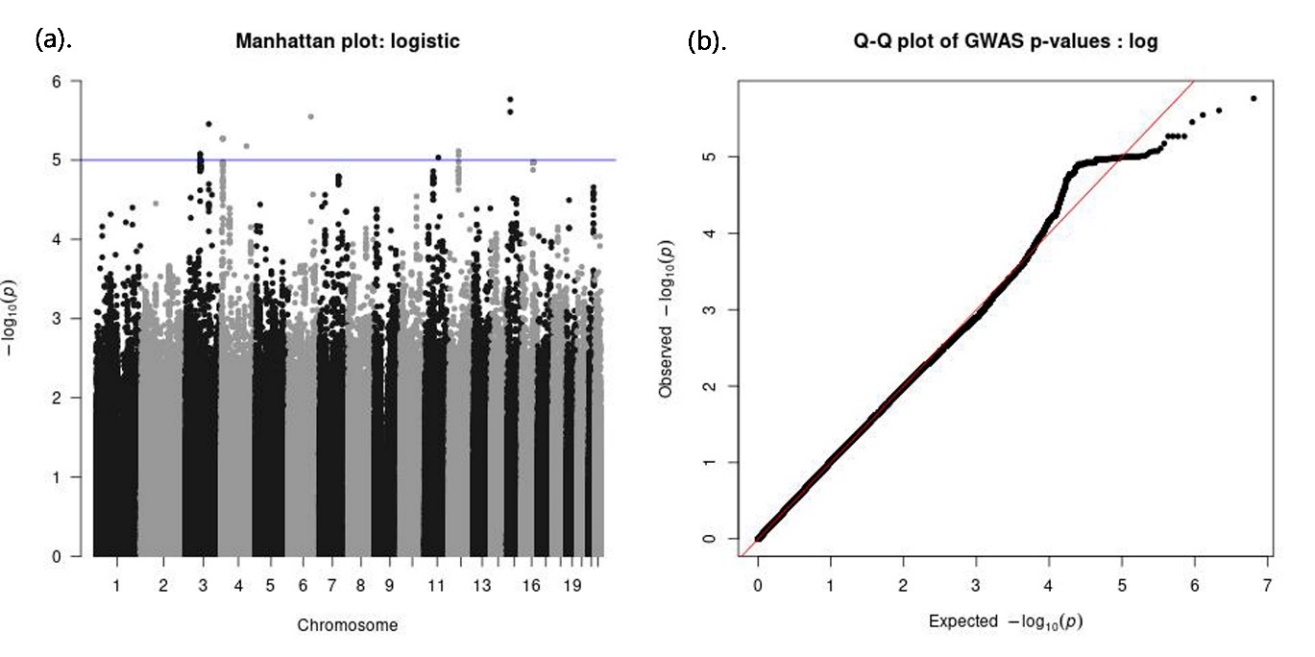


Figure.S6 Manhattan plot and Q-Q plot for legume intake frequency (GIF = 1.022)


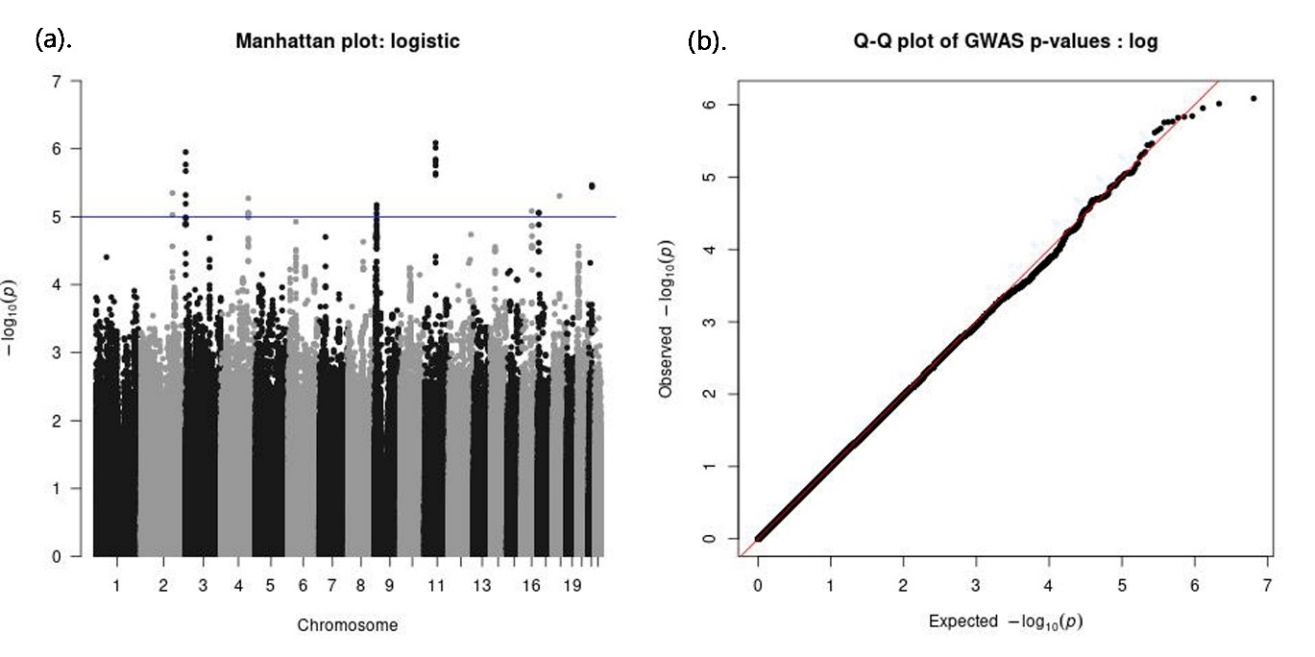


Figure.S7 Manhattan plot and Q-Q plot for meat intake frequency (GIF = 1.010)


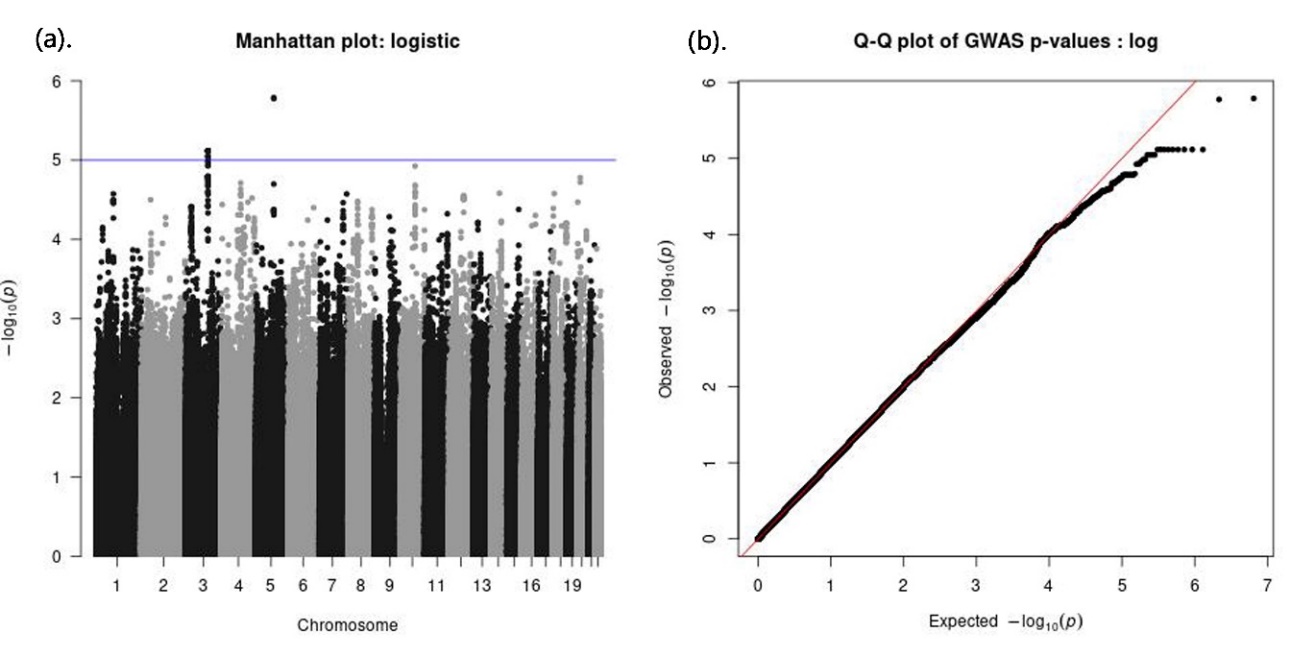


Figure.S8 Manhattan plot and Q-Q plot for sugar intake frequency (GIF = 1.031)


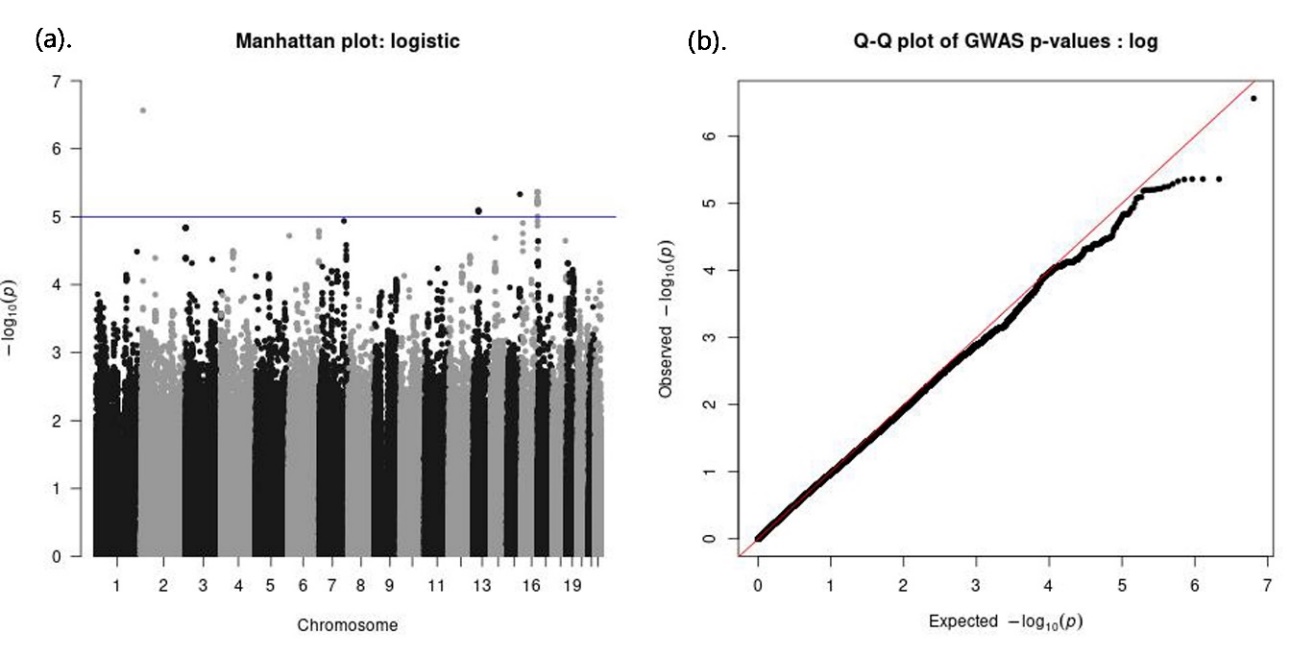


Figure.S9 Manhattan plot and Q-Q plot for vegetable intake frequency (GIF = 1.028)


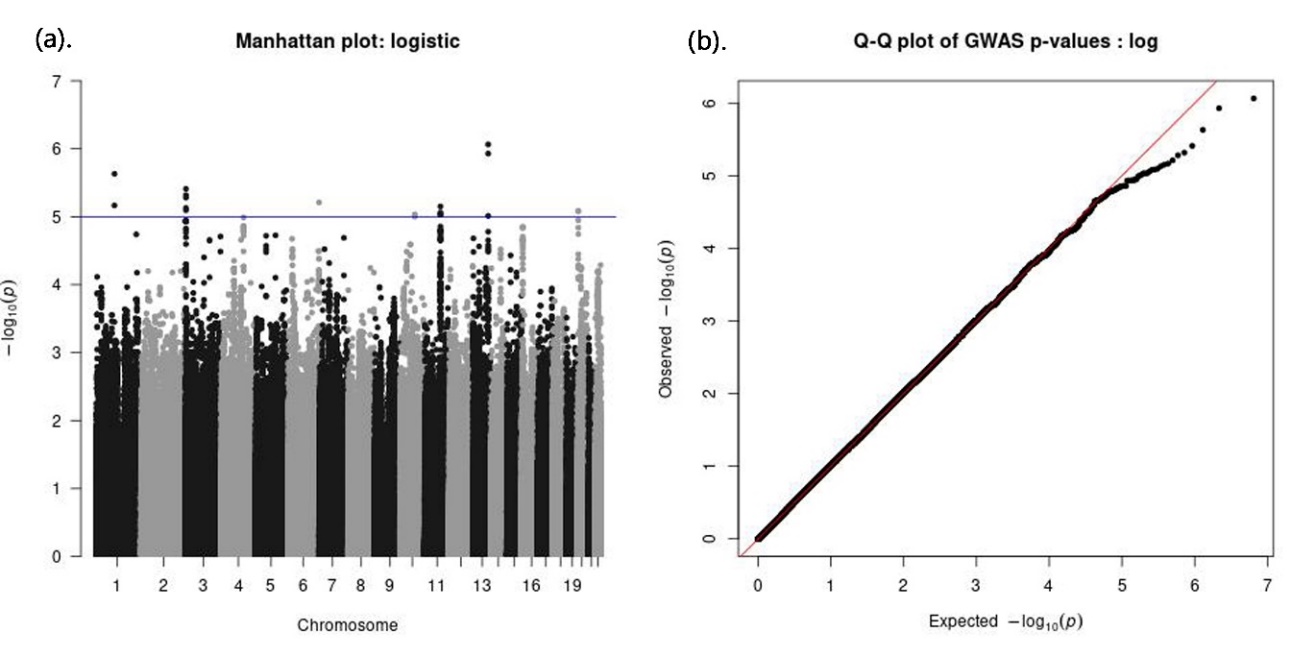


Figure.S10 Manhattan plot and Q-Q plot for exercise status (GIF = 1.017)


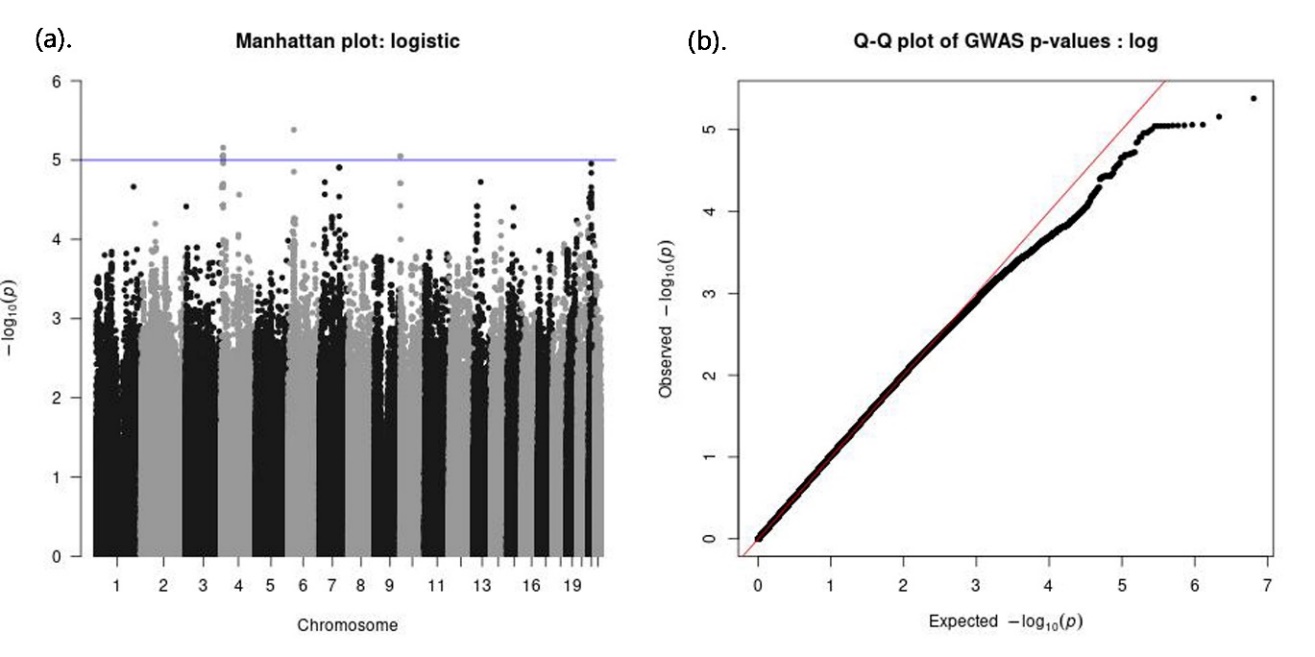


Figure.S11 Manhattan plot and Q-Q plot for housework (GIF = 1.034)


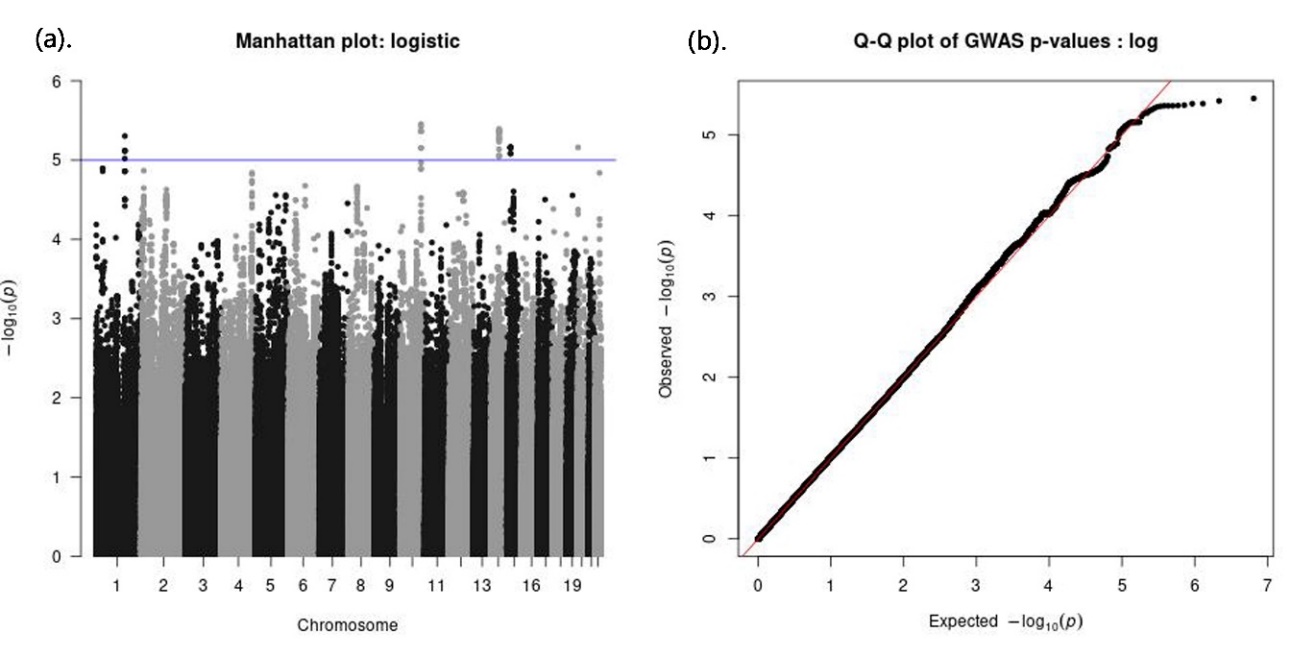


Figure.S12 Manhattan plot and Q-Q plot for mahjong (GIF = 1.029)


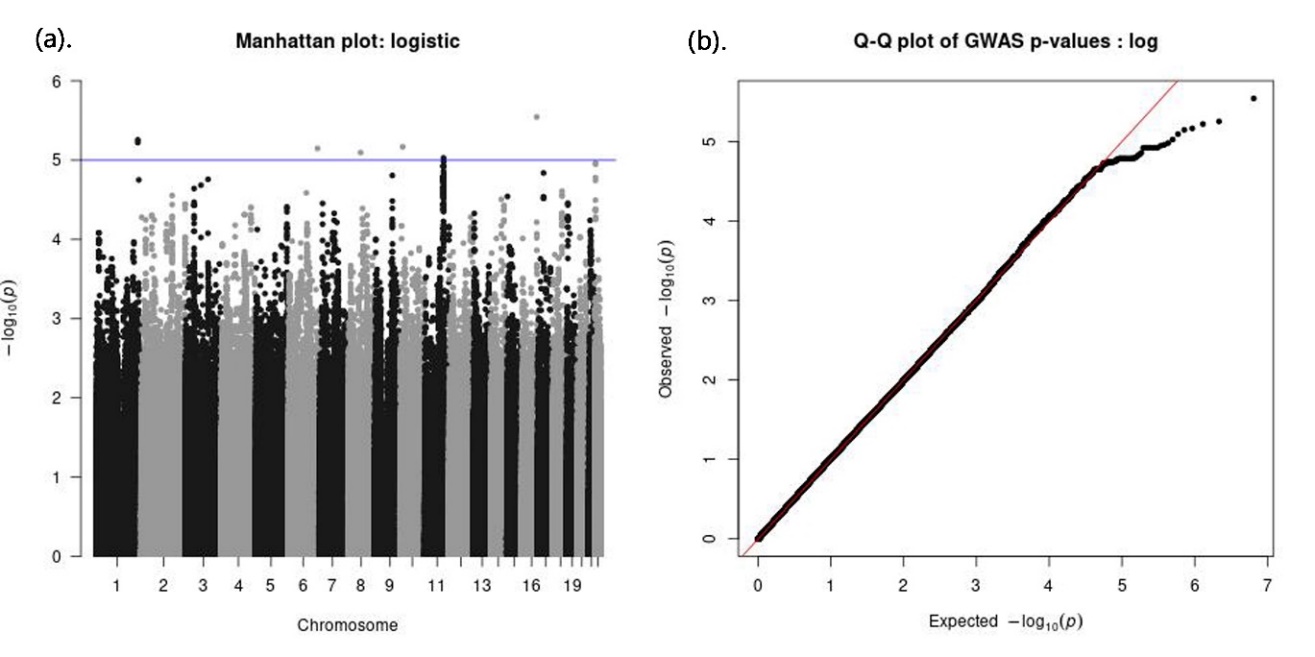


Figure.S13 Manhattan plot and Q-Q plot for open-air activity (GIF = 1.025)


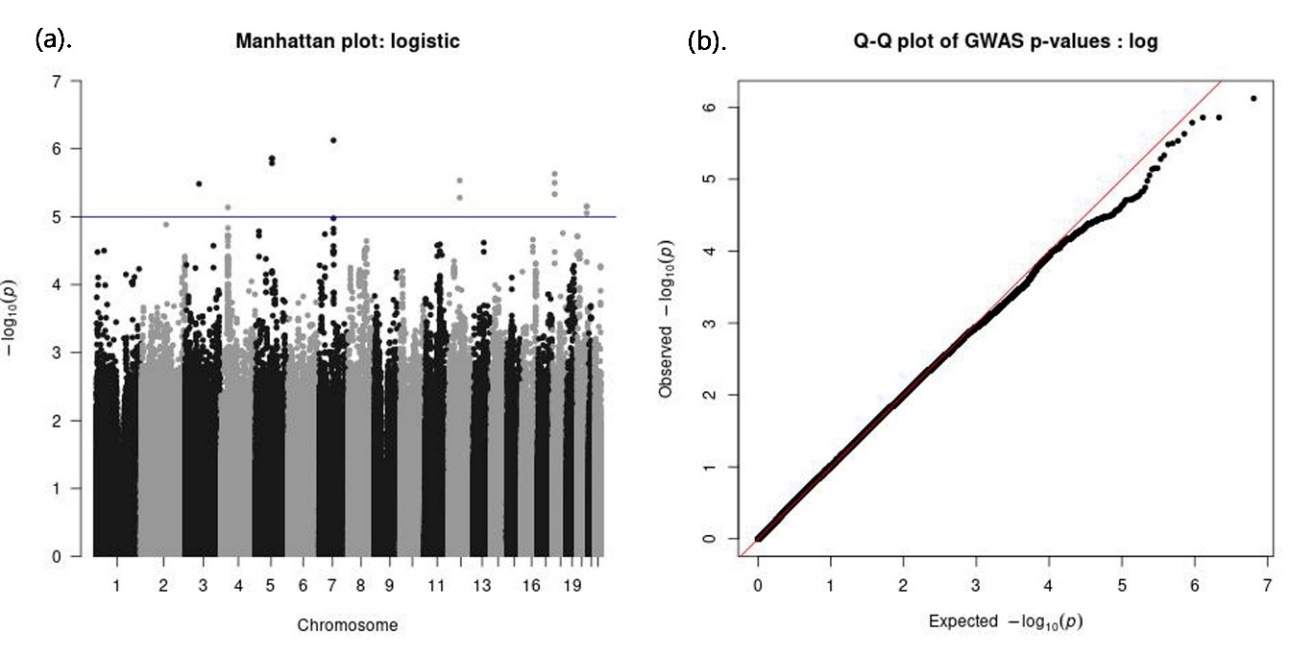


Figure.S14 Manhattan plot and Q-Q plot for pet ownership (GIF = 1.041)


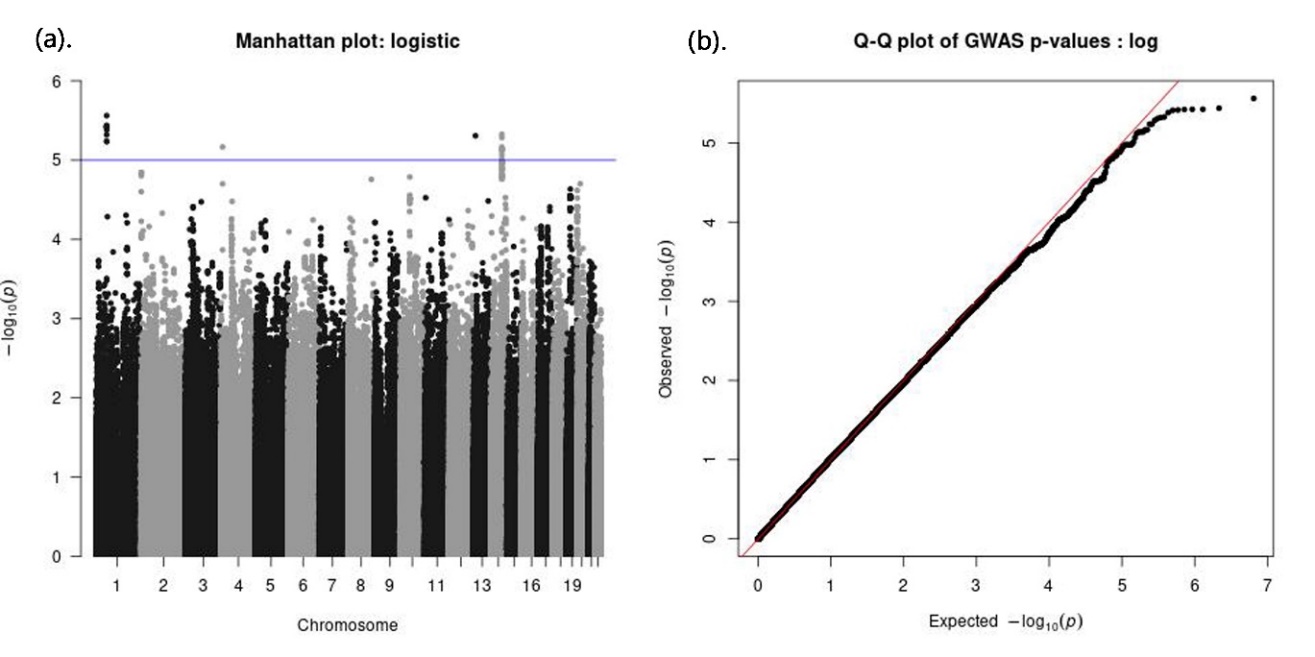


Figure.S15 Manhattan plot and Q-Q plot for reading (GIF = 1.036)


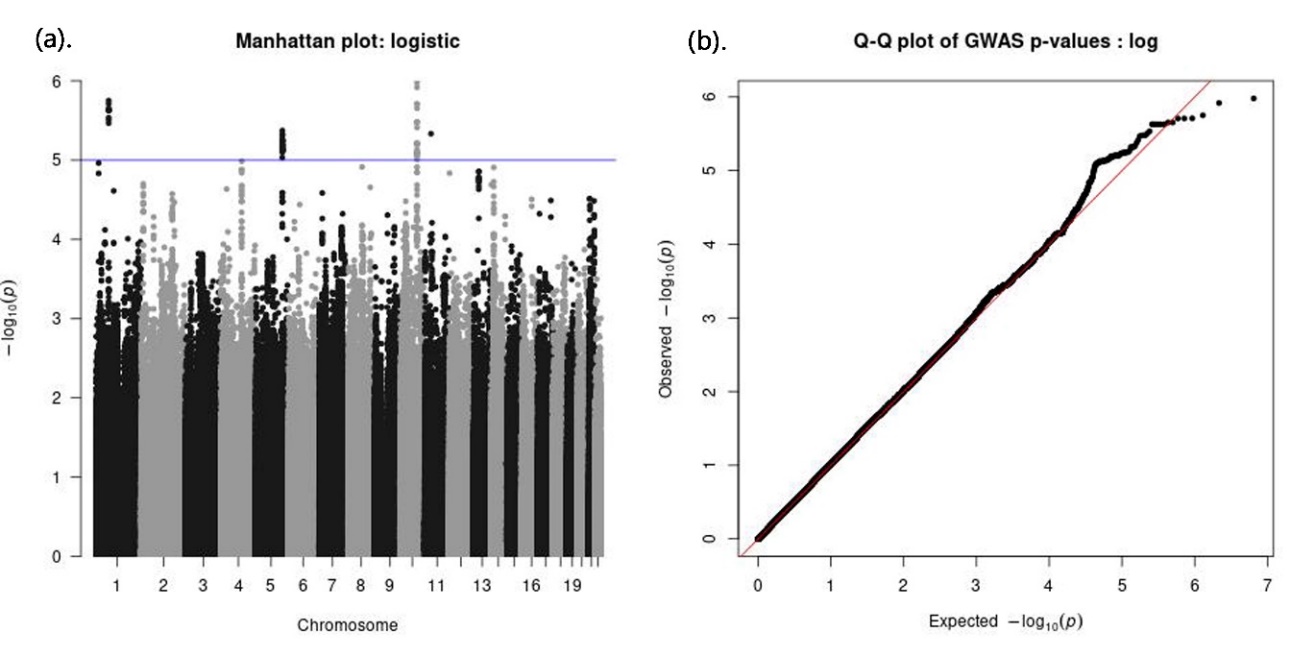


Figure.S16 Manhattan plot and Q-Q plot for TV/radio (GIF = 1.021)


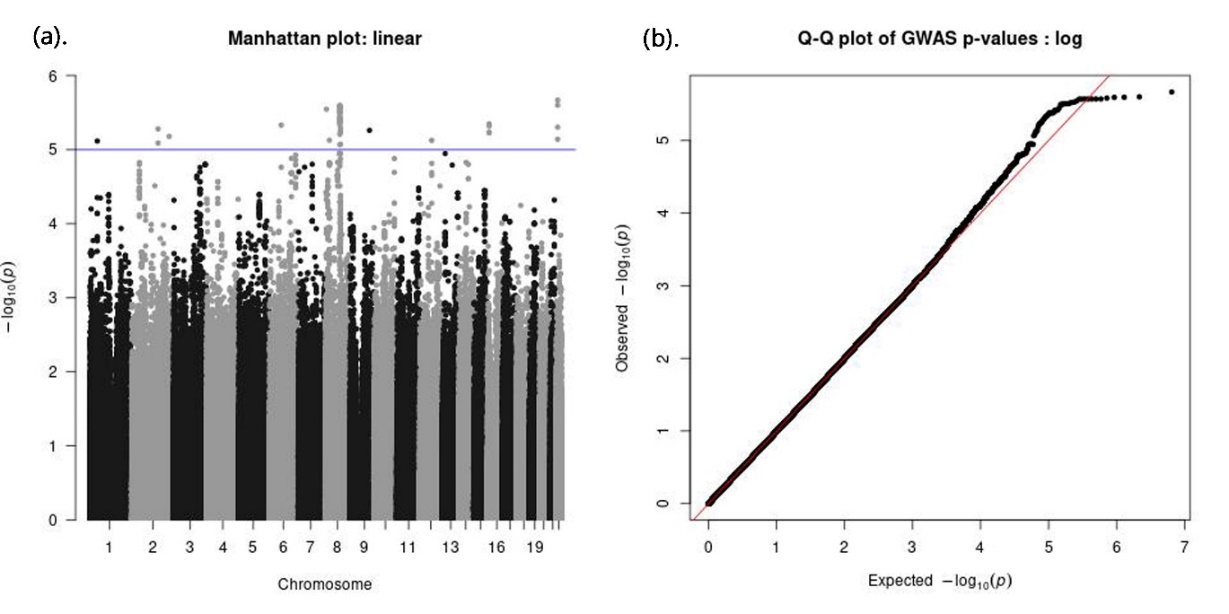


Figure.S17 Manhattan plot and Q-Q plot for education (GIF=1.006)


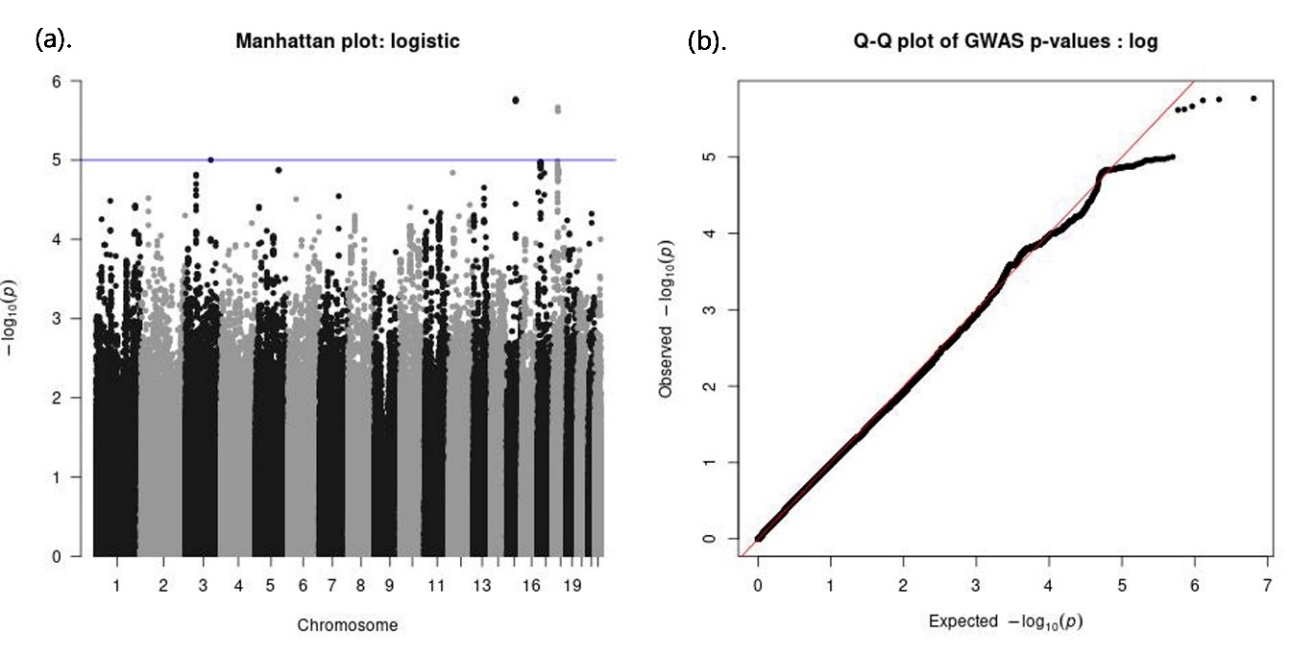


Figure.S18 Manhattan plot and Q-Q plot for stroke/CVD (GIF=1.018)
